# Novel Corona Virus 2019 Disease: Assessment on High-Resolution Computed Tomography Thorax

**DOI:** 10.7759/cureus.35506

**Published:** 2023-02-26

**Authors:** Anıl K Sakalecha, Varshitha GR, Sai Soumya Thati, Aashish Singh, Shantala Sawkar, Guru Yogendra Muthyal

**Affiliations:** 1 Radiodiagnosis, Sri Devaraj Urs Medical College, Kolar, IND; 2 Radiology, Sri Devaraj Urs Medical College, Kolar, IND; 3 Radiodiagnosis, Sri Devraj Urs Medical College, Kolar, IND

**Keywords:** computed tomography, crazy pavement, viral infection, pneumonia, ground glass opacities, corona virus, covid-19

## Abstract

Objectives

This particular study was undertaken to assess the role of high-resolution computed tomography (HRCT) thorax in diagnosing patients with novel Corona virus-2019 disease and screening suspected COVID-19 cases. It also involves an assessment of the severity of bilateral lung involvement in proven and suspected cases of COVID-19 infection.

Materials and methods

Two hundred and fourteen symptomatic cases referred to the department of radio-diagnosis were evaluated in this study. HRCT thorax was performed on SIEMENS Somatom Emotion 16-slice spiral CT. Initially, a tomogram was taken, followed by sections in the lung window at B90s, kVp 130, with a pitch of 1.15. The images are then reconstructed into 1.0-mm-thin slices. Radiologists then interpreted the scans for features of COVID-19 disease. Various imaging features and the severity of the disease were analysed in all patients.

Results

We observed that the male population was more affected by the disease (72% of the total cases). The most consistent and common HRCT finding is that of ground-glass opacity (GGO), which was present in 172 cases, corresponding to 78.4% of the cases. Crazy pavement appearance was seen in 41.2 % of the cases. Other findings included consolidation, discrete nodules surrounded by ground-glass opacification, subpleural linear opacities, and tubular bronchiectasis.

Conclusion

HRCT thorax plays an ideal role in diagnosing COVID-19 disease with high sensitivity and also provides prompt results as compared to RT-PCR. It also helps in grading the severity of the disease based on various patterns and the extent of lung parenchyma involved. Therefore, because of the immediate results and the ability to grade the disease, HRCT became invaluable in directing the treatment of COVID-19 disease.

## Introduction

In December 2019, an outbreak of novel coronavirus infection (severe acute respiratory syndrome coronavirus 2; SARS-CoV-2) occurred in Wuhan, Hubei Province, in the country of China. The coronavirus is an organism that can cause pathologies ranging from mild flu-like symptoms to severe conditions like acute respiratory syndrome (SARS) and Middle East respiratory syndrome (MERS) [1]. The virus is known to have human-to-human transmission. The number of novel coronavirus cases in 2019 showed an exponential increase around the world. Radiologists are interpreting more chest CT scans in those suspected of infection as well as in confirmed cases to ascertain the severity of the disease. Chest CT is an indispensable component in the diagnosis of patients suspected of having a COVID-19 infection. Indeed, given the limited number of reverse transcriptase polymerase chain reaction (RT-PCR) kits in some centers in India and false-negative RT-PCR results, some bodies have encouraged diagnosis based on clinical grounds and HRCT chest findings [2].

Out of the available laboratory tests, RT-PCR is the most specific and currently the gold standard for diagnosing COVID-19. Since RT-PCR takes a longer time to make the diagnosis, there was a need for an investigation with high sensitivity and quick results. CT imaging also plays a key role in the appropriate management of this infection. COVID-19 staging aids in determining whether a patient has COVID-19 infection prior to RT-PCR results or in the case of a false negative RT-PCR [3]. It also helps in assessing the progression and resolution of the disease after the initiation of treatment [4].

Review of literature

Since March 11, 2020, the World Health Organization (WHO) has declared Coronavirus disease 2019 (COVID-2019) caused by SARS-CoV-2 to be a pandemic and health emergency of international concern. A confirmed case is defined as a patient with RT-PCR test-proven COVID-19, irrespective of clinical signs and symptoms (asymptomatic), according to the WHO [5]. The first literature evidence of CT chest findings of COVID-19 infection was reported in January 2020. The main features include bilateral, subpleural, patchy ground-glass opacities (GGO) predominantly in the lower lobes, vascular enlargement, and posterior predilection, which were documented in several studies [6].

Typical HRCT findings in SARS COVID-19 cases

A wide variety of CT findings in COVID-19 are reported in different studies. However, all studies indicated that the main CT feature of COVID-19 pneumonia is the presence of peripheral, multifocal, GGO [7]. Multi-lobar involvement, predominantly in the lower lobes, is reported. These GGO may be interspersed with patchy areas of consolidation and intralobular-interlobular septal thickening with the classic ‘crazy paving appearance’. Linear consolidations and other signs that indicate organizing pneumonia, like the reverse halo sign (i.e., ‘areas of ground-glass opacities surrounded by peripheral consolidation’) may be rarely observed. In a study done by Salehi et al., the most common HRCT finding was GGO (88.0% of cases); consolidation was seen in 31.8% of cases with bilateral pulmonary involvement in 87.5% of cases; and most of the patients (about 76% of patients) had a peripheral distribution of GGO [8].

The other main differential for GGO is bacterial pneumonia, which is usually seen on HRCT as airspace consolidation with lobar or segmental involvement, limited by pleural margins including fissures. Additional common findings in bacterial pneumonia encountered on cross-sectional imaging are centrilobular nodules, cavities, and mucoid impaction, which are not commonly seen in COVID-19 infection [9].

Another differential diagnosis for GGO is an infection caused by Pneumocystis jiroveci pneumonia. Although ground-glass opacity is the main imaging feature, its distribution within the lung parenchyma is not similar to that observed in patients with COVID-19. It is predominantly perihilar, with a tendency to spare the subpleural regions. Since it occurs in immunocompromised patients, a detailed history of the patient’s immunity status should be known [10].

Pulmonary edema also has similar HRCT features, with GGO being mostly seen in the perihilar regions and giving a characteristic batwing appearance. Additional features such as cardiomegaly and signs of right heart failure can help in differentiating COVID-19 pneumonia.

Other findings

It is difficult to distinguish COVID-19 from pneumonia due to other viral causes. CT features overlap, even though it has been reported that CT abnormalities in COVID-19 pneumonia will more frequently exhibit a peripheral predominance. Less frequent findings in COVID-19 are pleural effusion and lymphadenopathy. COVID-19 infection should be suspected in cases presenting with respiratory symptoms and HRCT showing ground-glass opacities in the current pandemic situation [11].

## Materials and methods

Data collection

This study was approved by the ethical committee of RL Jalappa Hospital. In our study, we evaluated the HRCT findings in proven and suspected cases of COVID-19 infection from August to November 2020 (a period of four months) to identify the different CT patterns.

CT protocol of high-resolution chest

Images were obtained in the supine position. Technologists who performed the scans were provided with a PPE (personal protective equipment) kit. Scans were done from the level of the upper thoracic inlet to the upper pole of the kidneys with the breath-hold technique. The following parameters were used: tube voltage of 130 kVp, rotation time of 1.0 s, detector collimation width of 2 × 0.25, and slice thickness of 5 mm with 1 mm reconstruction. All images are then transferred to the workstations Myrian and Osirix for multiplanar reconstruction (MPR) and detailed evaluation.

Image analysis

Imaging findings were analysed by two experienced radiologists. The lesions were evaluated with respect to laterality, distribution, lobar involvement, presence or absence of consolidation, interlobular and intralobular septal thickening, peri-bronchovascular interstitial thickening, and other associated findings such as pleural thickening, pleural effusion, and mediastinal lymphadenopathy. The above imaging appearance was thoroughly analyzed and evaluated.

The severity and level of suspicion of COVID-19 infection in the patients are assessed based on CT severity and CO-RADS scores, which are calculated as explained below. In our study, we assessed all five lobes of the lungs separately (right lung: three lobes - upper, middle, and lower; left lung: upper and lower lobes) by a semi-quantitative method on CT, and a score of zero to five is given based on the amount of lobe of the lung involved (Table 1).

**Table 1:** Semi-quantitative assessment of percentage involvement of each lobe of lung.

Percentage of involvement of the lobe of the lung (%)	Score
0	0
<5	1
5–25	2
25–50	3
50–75	4
>75	5

The individual lobar scores were then added to calculate a total score out of 25 [12], based on which patient is grouped as having either mild, moderate, or severe pneumonia (Table 2).

**Table 2:** CT severity score. [12].

CT severity score	Severity of disease
0–7	Mild
8–15	Moderate
16–25	Severe

Based on the level of suspicion of COVID-19 infection in the patient, a CO-RADS score has been given [13] (Table 3).

**Table 3:** CO-RADS-level of suspicion of COVID-19 infection. [13].

		CT findings
CO-RADS 1	No	Normal or non-infectious abnormalities
CO-RADS 2	Low	Abnormalities consistent with infections other than COVID-19
CO-RADS 3	Indeterminate	Unclear whether COVID-19 is present
CO-RADS 4	High	Abnormalities suspicious for COVID-19
CO-RADS 5	Very high	Typical COVID-19
CO-RADS 6	PCR +	

Statistical analysis

Statistical analyses were done using Microsoft Excel (Microsoft® Corp., Redmond, WA). The statistical analysis was performed with the SPSS statistical package (version 21; IBM Inc., Armonk, USA) and OpenEpi (version 3.01). Measurable data were expressed as means ± standard deviations and categoric variables as counts and percentages. With the use of RT-PCR results as the reference standard, the sensitivity, specificity, and accuracy of chest CT were calculated.

## Results

Age and gender distribution

In this study, we evaluated a total of 214 symptomatic cases, of which 131 patients were RT-PCR positive for COVID-19. The remainder of the patients with suspected COVID-19 underwent HRCT of the thorax. The study included 155 males and 59 females. The mean age of the population was 49.5 years, with a standard deviation of ±15.9 years.

HRCT findings

**Figure 1:**
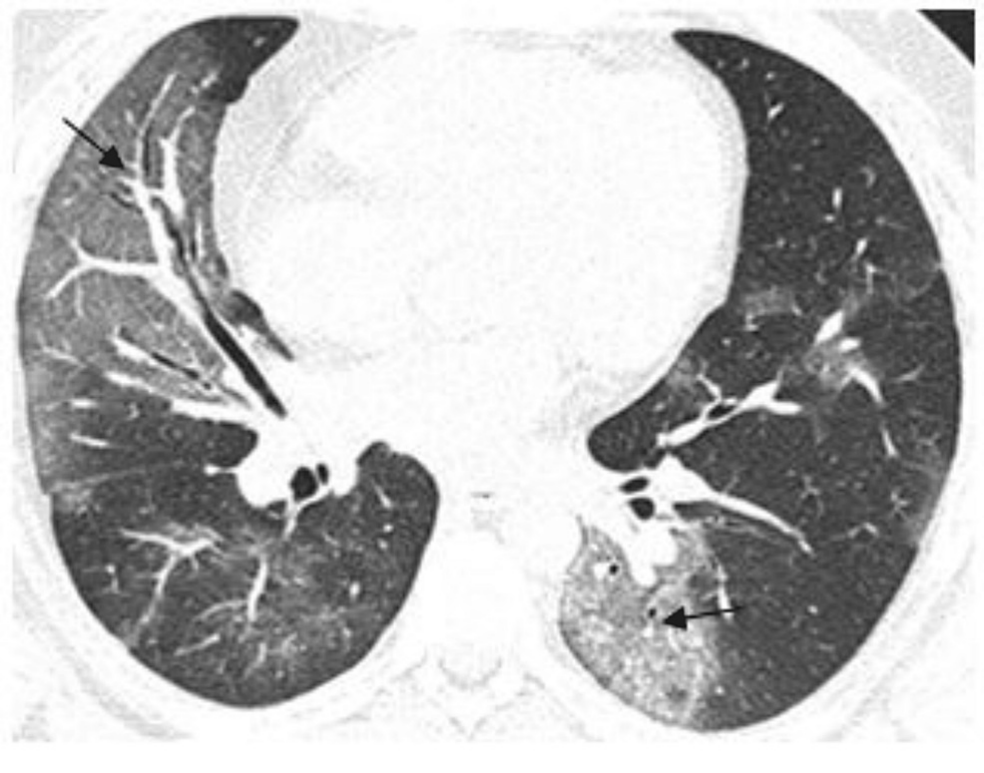
HRCT axial section of 38 Y/M COVID-19 positive patient showing ground-glass opacities with interlobular and intralobular septal thickening giving “crazy paving” pattern (arrows) in bilateral lung parenchyma.

**Figure 2:**
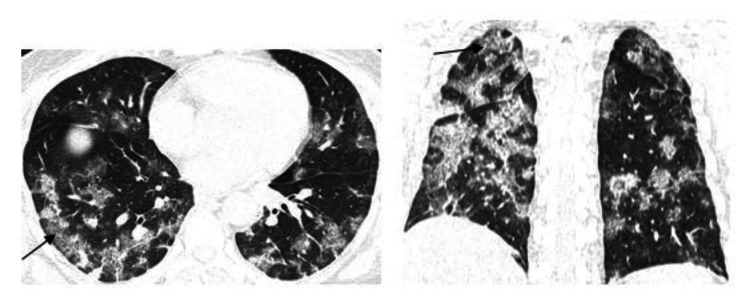
HRCT axial and coronal sections showing multilobar patchy areas of ground-glass opacities with interlobular and intralobular septal thickening giving “crazy paving” (arrows) pattern in bilateral lung parenchyma with few patchy areas of consolidation.

We observed that the most consistent and common HRCT finding is that of GGO, which was present in 172 cases, corresponding to 80.4% of the cases. In most cases, ground-glass opacities were superimposed with intralobular and interlobular septal thickening, which is known as the "crazy pavement appearance" (Figures 1-2).

This finding was seen in 89 cases, which is 41.2% of the cases. It is a non-specific finding, seen in pulmonary infections like bacterial, viral, Pneumocystis jiroveci, and mycoplasma, and also in a multitude of other conditions including pulmonary oedema, usual interstitial pneumonia (UIP), non-specific interstitial pneumonia (NSIP), pulmonary haemorrhage, alveolar proteinosis, acute interstitial pneumonia (AIP), organising pneumonia, and adult (acute) respiratory distress syndrome (ARDS). Multilobar peripheral involvement was seen in 70% of cases in our study. Bilateral involvement was seen in 74.2% of the cases (159 cases). A minor percentage of the patients had unilateral lung involvement (Table 4).

**Table 4:** Side of involvement.

Laterality	
Unilateral	16
Bilateral	159
HRCT negative for COVID-19 (but RT-PCR positive)	39
Total	214

About 39 patients (7%) who were RT-PCR positive, yet had no changes in both the lungs.

Other findings

**Figure 3:**
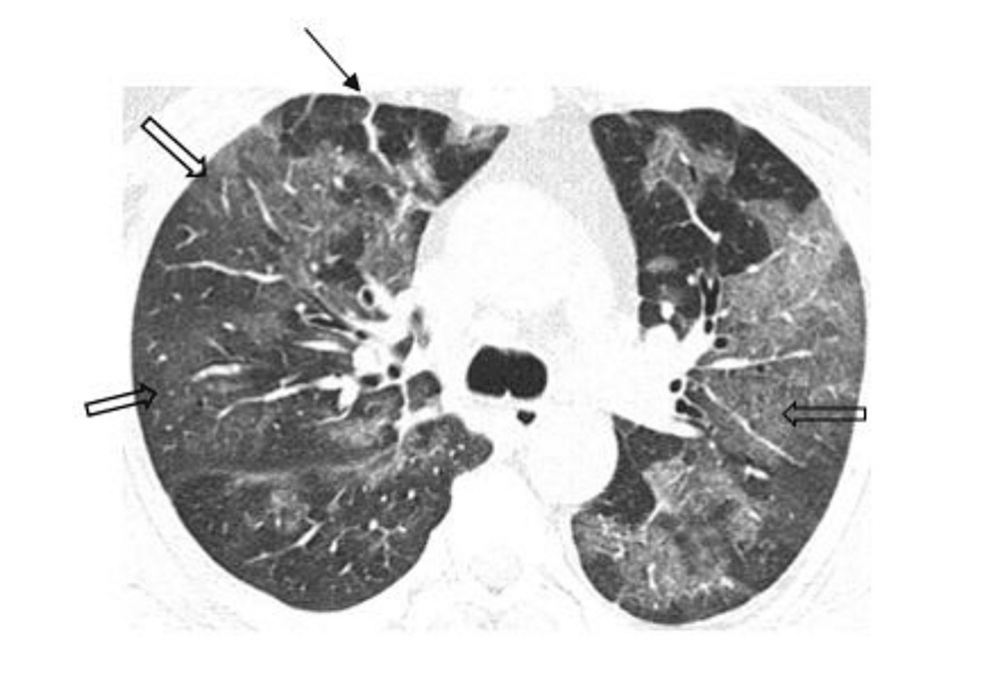
HRCT axial section of 48Y/F, COVID-19 suspect with CO-RADS 5 showing diffuse ground-glass opacities (block arrow) in bilateral lung parenchyma. Parenchymal/fibrotic bands (arrow) noted in medial segments of right middle lobe.

**Figure 4:**
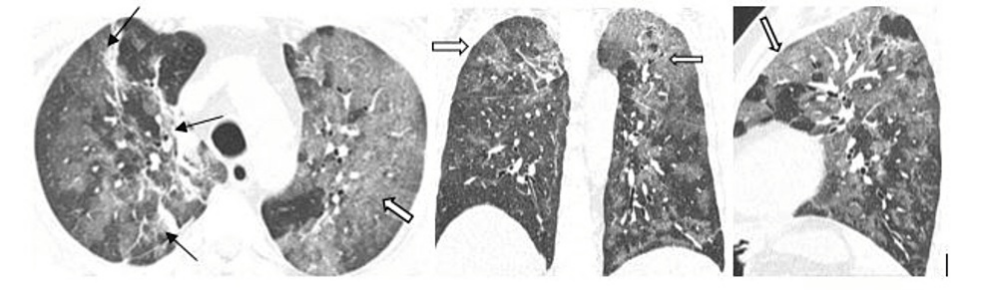
HRCT axial, coronal, and sagittal images of 56Y/M Covid-19 positive patient showing multilobar of patchy ground-glass opacities (block arrow) in bilateral lung parenchyma, crazy paving in left upper lobe, subsegmental atelectasis and parenchymal bands (arrow) in right upper lobe.

**Figure 5:**
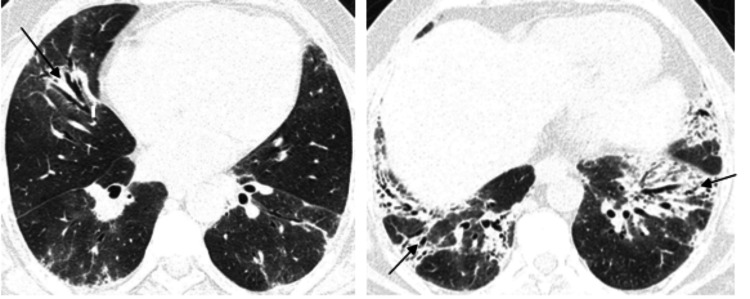
HRCT axial section showing peri-bronchovascular interstitial thickening (arrows) involving bilateral lower lobe, areas of consolidation and air bronchogram.

**Figure 6:**
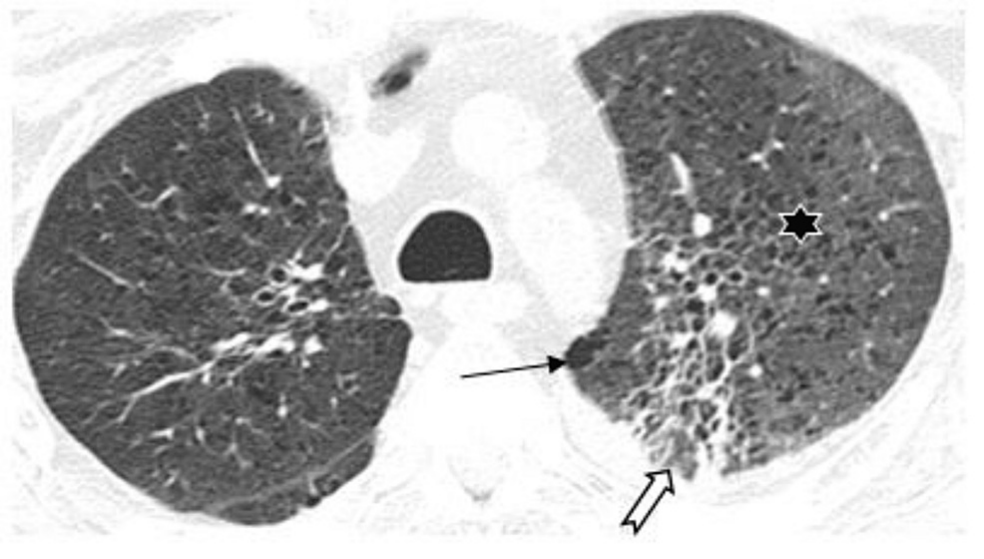
HRCT thorax axial section of 70Y/M patients with CO-RADS 5 showing centrilobular (star) and paraseptal (arrow) emphysematous changes in left upper lobe. Few parenchymal bands (block arrow) noted in apicoposterior segment of left upper lobe.

Other findings included subpleural linear abnormalities, parenchymal or fibrotic bands (Figure 3), consolidation with an air bronchogram, subsegmental atelectasis (Figure 4), and discrete nodules surrounded by ground-glass opacification seen in 10% of the cases. Peri-bronchovascular interstitial thickening was seen in 25 cases (11%) (Figure 5). Areas of paraseptal and centrilobular emphysema were seen in 11 cases, out of which 8 were males who had a prior history of smoking (Figure 6).

Bronchiectasis was seen in 48 cases (22%), which were of the cylindrical type in most cases. Few of the cases showed evidence of tractional and cystic bronchiectasis. Only 22 (10%) cases had pleural effusion, which signifies that it is an uncommon finding in the setting of COVID-19 infection. Evidence of pleural calcification was seen in three cases. Most of the cases showed no significant mediastinal lymph node enlargement.

The CT severity score and CORADS grading were assessed in all the patients. In our study, most of the patients had a mild COVID-19 infection, with ground-glass opacities being the most common finding in them (Table 5).

**Table 5:** Severity of infection among patients based on CT severity score.

CT severity score	Severity of disease	Number of patients
0 – 7	Mild	90
8 – 15	Moderate	64
16 - 25	Severe	60

Almost all the RT-PCR-positive patients (131 subjects) had positive findings pertaining to COVID-19 on HRCT. However, about 52 patients who were RT-PCR negative had positive findings corresponding to COVID-19 pneumonia on HRCT. Fifteen patients with mild symptoms had no findings on HRCT and were also negative for RT-PCR (Table 6).

**Table 6:** CORADS distribution of cases.

	No. of cases	Percentage %
CORADS I	15	5.1
CORADS II	4	1.8
CORADS III	8	4.6
CORADS IV	4	1.4
CORADS V	52	22.1
CORADS VI	131	61.2

Extra-pulmonary findings

A few extra-pulmonary findings were found in our study. Around 89% of the cases showed hyperdensity within the gall bladder, possibly sludge. This finding was not reported in other studies, to the best of our knowledge. 

## Discussion

After the outbreak in the city of Wuhan, China, the COVID 2019 infection became a pandemic with an exponential increase in cases with the progression of time. HRCT forms an important investigation in diagnosis and predicting the progression of the disease. It is widely available and performed worldwide. Hence, we have undertaken this study to establish the role of HRCT in the diagnosis and management of COVID-19 [5].

The HRCT patterns of viral pneumonia are related to the pathogenesis of infection. Viruses of the same family (Coronaviridae) have a similar pathogenesis. Therefore, viral pneumonia caused by different viruses from the same virus family presents with a similar pattern on chest CT images. SARS-CoV and MERS-CoV were identified as a part of the Coronaviridae family in 2003 and 2012, respectively. According to genome analysis, SARS CoV-2 belongs to the Beta-coronavirus genus [14]. Of the 214 patients in our study, only 42 (21.6%) had neither GGO nor consolidation. These results demonstrate that GGO and consolidation are the two main features of COVID-19.

Angiotensin-converting enzyme II acts as a key molecule in the development of acute lung injury and also plays a vital role in the severity of the progression of infection. Direct lung injury observed in SARS-COVID-19 infection is also induced by angiotensin-converting enzyme, which is an important contributing factor for diffuse alveolar damage. This may also explain the mechanism of sequential changes in CT findings, including GGO and consolidation [1].

Our results are consistent with the observed trend of having bilateral GGO or patches of consolidation. These findings should prompt radiologists to consider COVID-19 high on the differential diagnosis. We observed that most of the elderly patients had breathlessness during the acquisition of HRCT, mostly during end-inspiration, so the acquisition of appropriate CT images was difficult. Therefore, radiologists should pay special attention to differentiating GGO or small patches of consolidation from motion artefacts when reviewing CT, which may result in falsely labelling a study with a higher than actual CORADS score.

In our study of suspected and proven COVID-19 infection, the distribution of disease was peripheral GGO, multilobar, and predominantly confined to the lower zones of the lungs. The lack of children with COVID-19 in our sample group is consistent with a study by Dyun et al., where the authors of that study suggested that children might be less likely to become infected with SARS-CoV-2 than adults or, if infected, may show milder symptoms than adults [15]. Children were excluded from our study as they have milder symptoms compared to adults and also due to the high radiation exposure of HRCT.

As the severity of COVID-19 pneumonia increases, parenchymal abnormalities eventually involve atypical areas, including the central pulmonary zone and upper lobes. In our study, we observed that patients showed a predominantly peripheral/subpleural distribution of disease and that it was most commonly confined to the mid and lower lobes of the lung. The increasing areas of involvement and density of GGO/consolidatory patches indicated more disease progression.

The imaging diagnosis of viral pneumonia based on CT was available earlier than that based on RT-PCR. Few studies have proven that in cases of high clinical suspicion of COVID-19 pneumonia showing negative RT-PCR results, HRCT findings were suggestive of COVID-19 infection. CT has a high accuracy in diagnosing COVID-19 infection and may be useful as a standard method for the diagnosis of COVID-19.

We feel that even though we are a tertiary health centre, the sample size was one of the limitations of our study, despite the enormous caseload of COVID-19.

## Conclusions

Management of a pandemic requires a multi-modal approach and innovative and unconventional methods. HRCT can be used to diagnose COVID-19 patients early as compared to the RT-PCR test, which takes a few days for its results and also has a high rate of inconclusive results. In view of the limited resources for RT-PCR, it is essential to detect early, isolate, and treat patients based on HRCT, thus helping radiologists form a vital component in keeping the current pandemic in check.
